# Notes of Medical Experiences in India, Principally with Reference to Diseases of the Eye

**Published:** 1885-12

**Authors:** 


*Notes of Medical Experiences in India, principally with reference to Diseases of the Eye.* By S. E. MAUNSELL. Pp. 115. London: H. K. LEWIS. 1885.

This is a modest record of work of a high order, done under disadvantageous circumstances. That the writer, without skilled assistance of any sort, and under difficulties that would have disheartened most men, was able, in eleven working months, to perform 267 extractions of cataract among the natives of India, and have only 22 failures from all causes, is very geatly to his credit. His remarks are not entirely surgical, or even medical: personal experiences of travel and residence in regions little known help to fill the pages of a very pleasing and interesting little volume.

